# Leveraging virtual communities of practice for cancer control in Africa: experiences from the Africa Cancer Research and Control ECHO

**DOI:** 10.3332/ecancer.2025.1878

**Published:** 2025-03-20

**Authors:** Johnblack K Kabukye, Alice B S Nono Djotsa, Adedayo Joseph, Charles Muya, Benda N Kithaka, Mishka K Cira, Leshia Hansen, Annet Nakaganda

**Affiliations:** 1 SPIDER – The Swedish Program for ICT in Developing Regions, Department of Computer and Systems Sciences, Stockholm University, PO Box 7003, Stockholm, SE-164 07, Sweden; 2 Uganda Cancer Institute, Upper Mulago Hill Road, PO Box 3935, Kampala, Uganda; 3 VA HSR&D Center for Innovations in Quality, Effectiveness and Safety, Michael E. DeBakey VA Medical Center, Houston, TX 77030, USA; 4 Section of Health Services Research, Department of Medicine, Baylor College of Medicine, Houston, TX 77030, USA; 5 NSIA – LUTH Cancer Centre, Lagos University Teaching Hospital, Ishaga Road Surulere, Private Mail Bag, Lagos 100254, Nigeria; 6 Department of Radiation Biology, Radiodiagnosis & Radiotherapy, College of Medicine University of Lagos, Ishaga Road Surulere, Private Mail Bag, Lagos 12003, Nigeria; 7 Kenya Network of Cancer Organizations, KMA Plaza Apartments, Block E Suite 1.1, Mara Road, Upper Hill, PO Box 106383-00101, Nairobi, Kenya; 8 Kilele Health Association, Landmark Plaza, 13th Floor, PO Box 1627 - 00200, Nairobi, Kenya; 9 Center for Global Health, National Cancer Institute, 9609 Medical Center Drive, Rockville, MD 20850, USA

**Keywords:** cancer, Africa, project ECHO, telemedicine, community of practice, case study

## Abstract

**Background:**

The growing burden of cancer in Africa requires innovative approaches for enhancing the cancer workforce on the continent. Virtual communities of practice (VCoPs) are one such approach that can be utilised for continuous professional development, networking and sharing of cancer knowledge and best practices among different stakeholders.

**Objectives:**

To describe the experiences of a cancer VCoP, the Africa Cancer Research and Control ECHO, and illustrate short- and long-term outcomes that are relevant to enhancing the cancer workforce in Africa.

**Methods:**

We collected quantitative and qualitative data about the 2022/2023 Africa Cancer ECHO through (i) statistics from the video conferencing platform (number of participants per session) and (ii) a cross-section survey at the end of the curriculum year in June 2023 (participants’ feedback about the sessions, learning and use of the knowledge and network from the ECHO). We also compared these data with evaluations of the ECHO from previous years, and conducted interviews with core community members to understand long-term outcomes regarding professional networking and collaboration.

**Results:**

The African Cancer ECHO has been running since 2018. Members meet regularly online to discuss different aspects of cancer control through didactical and case presentations. In 2022/2023, twelve 90-minute monthly sessions were held with an average of 33 attendees per session and 200 unique individuals from 14 African countries overall, plus additional representation from outside of Africa.

Over the 4 years (2019/2020 to 2022/2023), different cancer control stakeholders participated in the Africa Cancer ECHO, including advocates, patients, clinicians, researchers and cancer planners. For each year, about 30% of the participants were new to the ECHO. The respondents were positive about the ECHO sessions and in agreement with the ECHO’s learning and networking outcomes 70%–90% of the time. The interviews revealed long-term, practical outcomes of the Africa Cancer ECHO, including securing research funding, initiation of a new community of practice to specifically address cancer survivorship in Africa and scientific research collaborations resulting in at least six peer-reviewed publications and several conference abstracts.

**Conclusion:**

VCoPs, such as the Africa Cancer ECHO, have the potential to contribute to human resource capacity-building for cancer control in Africa through accessible and convenient peer learning, professional networking and collaboration. However, the identification and use of more robust evaluation tools and methods may provide a more comprehensive assessment of all of their benefits, including short and long-term outcomes.

## Introduction

The growing cancer burden in Africa has been highlighted by several reports recently, with cancer cases and deaths predicted to double over the next decades – reaching 2.1 million new cancer cases and 1.4 million cancer-related deaths by 2040 [1–6]. One of the challenges to cancer control in Africa is the severe shortage of the cancer workforce. Generally, the number of health workers in Africa is low compared to other regions of the world. A recent WHO survey showed that there are approximately 1.55 workers (physicians, nurses and midwives) per 1,000 people [7] compared to 3.9 doctors and 8.4 nurses per 1,000 people in Europe [8] and well below the WHO threshold of 4.45 health workers per 1,000 people [7]. This shortage is even more pronounced for cancer care providers. For example, a review by Trapani *et al* [9] found that there were 0.27 clinical oncologists per 100 cancer patients globally, while in Africa, this figure stood at 0.01, with some countries having none at all. African oncologists report higher workloads and lower job satisfaction compared to elsewhere in the world [10].

Efforts to increase the cancer workforce in Africa are urgently needed. Training and retention of oncology specialists (medical, surgical, radiation oncologists, pathologists and so on) should be bolstered, as should the capacity building of frontline non-specialists, such as primary care providers, nurses and community health workers. These frontline healthcare workers provide cancer education, screening and prevention. They are often patients’ first point of contact, making them essential for early diagnosis and referral, particularly in rural communities. Additionally, engagement and involvement of policy-makers, patients and patient advocacy groups and the general public is needed to disseminate knowledge and garner action from all cancer control stakeholders.

Virtual communities of practice (VCoPs) [11, 12] offer a more accessible, affordable, flexible, multidisciplinary and inclusive alternative for capacity building of the cancer workforce compared to traditional medical education. This is particularly relevant to low and middle-income countries (LMICs), such as in Africa, given the increasing access to the internet and video-conferencing technologies and the extra push for telemedicine and distance learning due to the COVID-19 pandemic. The Africa Cancer Research and Control ECHO is one such VCoP. Adapted from the highly successful Project ECHO^®^ by Arora *et al* [13], the Africa Cancer ECHO facilitates the sharing of practical experience by cancer control practitioners, including non-clinical stakeholders such as patients, advocates and policymakers, usually from different geographical locations, using web-conferencing tools. This peer-to-peer learning and networking allows the sharing of contextually appropriate cancer control initiatives, cross-fertilisation of ideas, continuous medical education through didactic and case-based learning, mentorship and opportunities for collaboration and career growth.

The Africa Cancer ECHO was first convened by the US National Cancer Institute in 2018. In 2019, a formal Steering Committee, led by former participants in the program, took over leadership of the program. It is convened with the aim of increasing familiarity with and utilisation of national cancer control planning (NCCP) principles and strategies, as well as strengthening interactions and collaborations among stakeholders in cancer control in Africa. Arora’s *et al* [13] original ECHO involved clinical management of a particular condition, such as hepatitis C, with specialists at an academic medical center (the hub) collaborating and mentoring non-specialists in community health facilities (the spokes) [13]. The Africa Cancer ECHO adapts the original ECHO model as it does not have a single academic center as a hub or affiliated community cancer care facilities as spokes. There are also no patients with a specific cancer being managed by non-specialists who are mentored. Instead, the Africa Cancer ECHO facilitates knowledge-sharing amongst individuals and institutions working in cancer care and research in Africa and beyond, including researchers, clinicians, patients and patient advocates, ministry of health cancer planners and other policymakers, funding agencies and so on.

The curriculum is derived from input by participants. Starting in 2022, the curriculum follows the NCCP core elements as outlined in Oar *et al* [14]. When adapting ECHO from a patient-centric care model to a population health model, ‘cases’ were redefined as ‘system or organisational contexts’ reflecting a larger unit of concern and target for adaptation. Patients undergoing cancer screening, diagnosis or treatment are presented during these parts of the curriculum (often by cancer specialists), but these serve an illustrative purpose to concretise and contextualise the discussions, as opposed to the traditional ECHO format where such case presentations are made by non-specialists who are then guided on how to manage the cases. Case studies of programs such as patient navigation, cancer advocacy initiatives, cancer screening programs or efforts to improve access to cancer medicines through price negotiations are also presented.

Evaluations of the Africa Cancer ECHO were conducted in 2018 [15], 2019–2020 [16, 17] and 2020–2021 [18]. The findings showed that participants’ experiences have been generally positive, with the ECHO perceived to facilitate learning and knowledge sharing, networking and the general cancer control discourse. There have also been reviews evaluating the outcomes and impact on patient care of ECHO and ECHO-like VCoPs in different medical conditions [19–21] and cancer in particular [22]. Across all these reviews, Project ECHO^®^ and similar VCoPs have been reported to have generally positive results on attendees (healthcare providers), patient advocates and patients. A recurring limitation, however, has been the low strength of evidence, with many evaluations being retrospective, non-experimental, subjective (self-) reports by attendees and with small sample sizes. Moreover, most of the assessments have focused on process measures and lower levels of Moore’s continuous medical education evaluation framework [23], i.e., on participation (e.g., numbers and types of attendees), satisfaction (e.g., how well the ECHO content met attendees’ expectations) and knowledge acquisition. More objective measures and measures at higher levels of Moore’s framework, i.e., application of acquired knowledge and changes in clinical outcomes of patients and communities, have been rarely studied. Where prospective studies have been done, the follow-up periods have been short, making it difficult to measure the long-term outcome of these capacity-building initiatives. This is especially relevant for models like the Africa Cancer ECHO, where outcomes do not relate to individual patients but rather are systemic and general and, therefore, take long to become evident (e.g., reduction in late-stage diagnosis or incidence of cancers). Finally, other potential outcomes or benefits of VCoPs, such as professional networking and collaboration, have rarely been examined.

Therefore, this paper aims to summarise the experiences of the 2022–2023 Africa Cancer ECHO, compare with experience from previous years, and highlight some of the distal outcomes relating to ***interprofessional networking and collaboration***. This provides a more comprehensive program evaluation, including benefits beyond knowledge acquisition and clinical care of individual patients. It also allows comparison and assessment of evidence strength.

## Methods

### Structure of the Africa cancer ECHO

The Africa Cancer ECHO is led by an all-volunteer steering committee consisting of cancer professionals from different African countries, who are responsible for identifying presenters and moderating the sessions. The Kenya Network of Cancer Organisations (KENCO) in Nairobi, Kenya, hosts the sessions via the video conferencing platform Zoom**^®^**. For the 2022–2023 period, sessions were held monthly and lasted 90 minutes. The structure was similar to the previous years, with presentations of ‘cases’ relevant to the topic (not a clinical patient case, but rather cancer control program interventions or programs) followed by didactic presentations and then open discussion by the participants.

### Data collection

Evaluation of the 2022–2023 period followed a similar method as the previous periods [15–18] as follows: (i) data about the sessions was routinely collected, including the topic, presenters’ details and number of participants; (ii) a survey of the attendees about their experience and opinions was conducted. The survey included both quantitative and short qualitative questions based on Kirkpatricks model of learning and training [24], which covers participants’ ***reaction*** to a program (opinion about the topics, speakers and session structure), ***learning*** from it (if they feel they gained new knowledge), change in ***behaviour*** (application of what they learned) and any business ***outcomes*** following the program (such as interpersonal networks and collaboration). Participants were also asked about any challenges and recommendations. The survey was distributed via email, including two weekly reminders, to the full Africa Cancer ECHO distribution list at the end of the 2022–2023 curriculum in June 2023.

In addition, we conducted five key informant interviews to explore the benefits and outcomes of the ECHO, particularly those beyond knowledge acquisition. The interview participants were purposively selected to include previous Africa Cancer ECHO steering committee chairpersons or other core members. We asked them why they remained committed and continued participating in the ECHO over the years to understand perceived long-term, high-level benefits beyond the individual session content. Field notes were made by the interviewer (first author) summarising the key themes or concepts related to the research question. Discussion of the findings from the interviews and constant comparison with the survey findings was done by all the authors in regular monthly meetings over 6 months.

### Analysis

Session details and quantitative survey results were summarised and tabulated as descriptive statistics, and a graph was created for visualisation. Reported networking and collaboration outcomes or initiatives were summarised into short narrative reports.

### Ethics

No review by a research ethics committee was sought as this evaluation posed a minimal risk to participants, i.e., no personally identifying information was collected, and participants were not patients under our care or other vulnerable groups. Online forms were used for data collection, and respondents were presented with a preamble explaining the purpose and use of the collected data and had the opportunity to consent or refuse to participate. There were no incentives given to participants.

## Results

### Summary of 2022–2023 sessions

Twelve 90-minute sessions were held with an average of 33 attendees per session (range 26–48). Overall, there were 200 unique individuals over the 12 sessions, with a third of these attending at least two sessions. Table 1 shows the topic and presenters’ details.

### Characteristics of survey respondents

The 2022/2023 evaluation survey was completed by 38 out of the 200 contacts on the distribution list who were invited, for a response rate of 19%. The respondents came from at least 14 African countries, with Kenya being the most represented with 13 respondents (34%). One respondent came from outside Africa (USA). The respondents reported working in advocacy or Cancer NGOs (13 respondents, 34%) or were clinicians, such as nurses and doctors (12 respondents, 32%), while the rest were researchers, healthcare administrators or patients. The majority (30 respondents, 79%) were returning members from the previous years of the ECHO.

Comparison with previous years showed similar overall characteristics, with 38, 22 and 37 respondents for the previous three surveys. They were from 9 to 13 different African countries, mostly in Anglophone Africa, and had comparable job roles, membership and attendance histories. Appendix 1 shows the rest of the participant details and survey responses.

**Figure 1.**
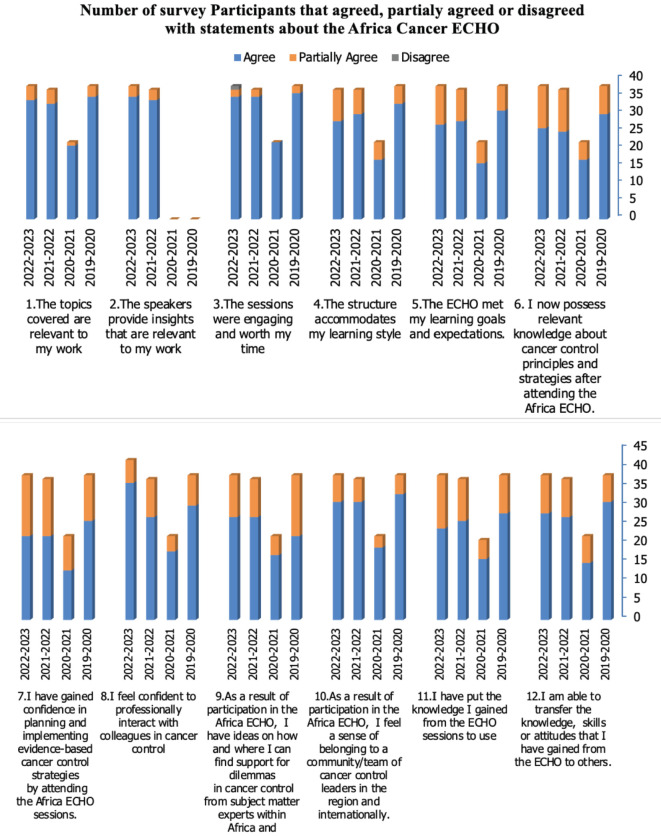
Number of survey respondents that agreed or disagreed with statements about the Africa cancer ECHO. Statements 1–5 concern reaction, 6 and 7 learning, while 8–12 concern behaviour and outcomes such as networking and community belonging [24].

Figure 1 shows participants’ responses to different statements about the Africa Cancer ECHO and its outcomes for years from 2019/2020 to 2022/2023. The respondents were positive about the sessions, with over 90% in agreement that the topics were relevant, the speakers insightful and the sessions engaging and worth their time. They also agreed that the ECHO structure aligned with their learning styles (≈80%) and that the ECHO met their expectations (≈75%). The respondents also indicated that the ECHO provided them with relevant cancer control knowledge (≈73%) and confidence in planning and implementing cancer control programs (≈61%), that the ECHO enabled professional networking regionally and internationally (≈82%), a sense of belonging or community (≈85%) and that they could find support when faced with dilemmas in cancer control (≈70%). Finally, respondents indicated that after attending the Africa Cancer ECHO, they had applied the knowledge gained (≈70%) and could pass it on to others (≈74%). These findings were also echoed in the qualitative comments in the survey, where respondents indicated that the most important reasons for them to participate in the ECHO were related to professional development and learning, as well as networking and collaboration.

The 2022/2023 survey respondents also indicated which obstacles they face when attending the Africa Cancer ECHO, with competing commitments being the most cited (26 respondents), followed by internet connectivity issues (13 respondents), the timing of the session (day and time) (13 respondents), the sessions being too long (2 respondents) and topics being irrelevant (1 respondent). This ranking of obstacles is similar to the previous years (Appendix 1).

### Narrative reports of collaboration and networking outcomes

#### Funding mobilisation: the Digital Africa Network for Cervical Cancer Elimination (DANCCE) initiative

The professional network, trust and shared goals built from participating in the Africa Cancer ECHO have resulted in links to funding sources and collaboration on grant applications and project execution to address important issues in cancer control in Africa. An example of this is the DANCCE initiative https://dancce.africa, which was funded by SPIDER https://spidercenter.org/. Africa Cancer ECHO hosts KENCO were linked to SPIDER by one of the members and case presenter from 2022, and subsequently, funding support was provided for KENCO to carry out a mapping of digital resources and initiatives in cervical cancer control in Africa. In addition, SPIDER funded Africa ECHO members from Uganda to implement and evaluate an integrated electronic cervical cancer screening registry and follow-up system at the Uganda Cancer Institute and its satellite clinics in 2023. This work resulted in two conference abstracts and a workshop at the 2023 AORTIC conference in Dakar, Senegal. In addition, manuscripts are under preparation, and discussions among ECHO members are being held to mobilise more funding to keep DANCCE activities going.

#### New communities of practice and initiatives for cancer control: Africa survivorship working group

In September 2020, the Africa Cancer ECHO held two sessions focused on cancer survivorship, one focusing on the clinical viewpoint and another on the patient or survivors’ viewpoint. Following the robust discussion and interest in this issue, a sub-group of participants set up separate meetings over the following months and eventually formed an (informal) Africa Survivorship Working Group. This group now includes 62 individuals who work in cancer survivorship research and patient support, as well as those with lived experience. The group has published a peer-reviewed article that examined the inclusion of cancer survivorship-related strategies and objectives in NCCPs of African countries [25] and an associated set of Survivorship Country Profiles made publicly available for policy decision-making on the portal of the International Cancer Control Partnership [26]. Demonstration of the policy impact of this engagement is the fact that the Nigeria Federal Ministry of Health was able to use the results of the study to include, for the first time, a Survivorship Pillar in the recently updated National Strategic Cancer Control Plan 2023–2027 [26] which will now guide strategic response to the needs of cancer survivors at the national level.

A second study by the survivorship working group, which is under review for publication at the time of writing the paper, is a scoping review of survivorship care in Africa. This study included research training and capacity building, as well as research leadership opportunities for research fellows and regional experts. It has also led to several conference presentation opportunities and discussions about the need for more support for the field of cancer survivorship research and writing that is led by cancer survivors about their lived experiences.

The survivorship group continues to conduct joint projects to understand the current landscape of cancer survivorship in Africa from the patient, clinical and policy levels to strengthen and expand cancer survivorship services and identify areas of focus for further context-specific research.

#### Platform for communication and networking

Another important outcome of the Africa Cancer ECHO from a professional networking perspective is that it provides a trusted social network where members share information and connect with cancer professionals and other stakeholders with contextual knowledge and interest in cancer control in Africa. For example, in December 2022, KILELE Health, in collaboration with FIND – the global diagnostics alliance, set out to gain an understanding of the landscape of secondary prevention of cervical cancer in Africa, particularly eastern Africa, where the burden is highest. Through the Africa Cancer ECHO, KILELE Health was able to secure contacts and connections with Civil Society Organisations (CSOs) working in Africa on cervical cancer and/or HIV/AIDS and subsequently established the Africa Cervical Health Alliance (ACHA), a network of 22 cancer CSOs from 15 African countries. The ACHA conducts targeted advocacy, capacity building and training, communication and engagement and resource mobilisation for community action toward cervical cancer elimination.

Similarly, KENCO used Africa Cancer ECHO’s online platform Basecamp https://basecamp.com/ (276 members) as one of the sources of information and participants for the cervical cancer digital resources mapping under the DANCCE initiative mentioned above. Members of the ECHO also use Basecamp to share funding opportunity announcements, upcoming conferences, job adverts, recent publications about cancer and distribution of research surveys or recruitment of research participants.

#### Joint publications

Africa Cancer ECHO members have collaborated on scientific manuscript writing about the ECHO itself and other topics, with at least five peer-reviewed journal publications and several conference abstracts and presentations. Table 2 summarises the journal articles.

## Discussion

In this paper, we evaluated a VCoP, the Africa Cancer ECHO, reviewing its outcomes for the 4 years 2019/2020 to 2022/2023. We triangulated data from different perspectives, including participation statistics, participant surveys and narrative reports from members, to increase the rigor and validity of the findings. The findings show that the Africa Cancer ECHO is a vibrant community of practice that facilitates peer learning, networking and collaboration among African cancer control stakeholders from many countries, leading to meaningful and practical outcomes that contribute to improvements in cancer control. It attracts new members while retaining its core members and maintaining its structure and identity. The members appreciate the value of being part of the community, and many consistently return and remain engaged despite challenges such as other commitments.

Perhaps the most significant findings and addition of this paper to evaluations of the Africa Cancer ECHO and similar VCoPs, are the long-term and practical outcomes highlighted in the narrative reports, i.e., funding opportunities, scientific collaborations and publications and new communities of practice. These outcomes, particularly those related to professional networking, are not often assessed [19–22], likely due to difficulty quantifying and attributing them to the ECHO. The survey questionnaires often used in evaluating ECHO, as well as the short evaluation period (e.g., at the end of an ECHO curriculum period), often miss out on these tangible outcomes. In this study, we asked core members of the Africa Cancer ECHO, who have been members of the VCoP for a long time, to reflect and indicate how being part of the community directly contributed to tangible outcomes.

Wenger *et al* [11] discuss value and value creation stories, where they assert that the value of the community (of practice) should be seen or assessed primarily from the perspective of the members and then secondary from other stakeholders such as sponsors or the organisations that members belong to. Furthermore, value is derived in the short and long term across different cycles or levels (see also Kirkpatrick’s model [24]); therefore, value creation stories should be used in addition to proxy data or indicators to minimise assumptions about the value of the VCoP and corroborate the indicators and causal attribution from the members. This supports our approach of using narratives from the members, which gives a nuanced understanding of how they derive value from the Africa ECHO beyond session attendance.

Nonetheless, in the absence of rigorous experimental design methods to account for confounders, attributing the outcomes to the ECHO remains difficult, both for short and long-term measures. For example, in the evaluation of the HIV ECHO in Namibia [27], it was not possible to distinguish the increase in provider knowledge, satisfaction and self-efficacy due to the ECHO or a concurrent clinical mentorship by the Ministry of Health.

Besides the ‘tangible’ outcomes, professional networking has other ‘soft’ benefits. Waters *et al* [28] argue that ‘the strength of the ECHO model is in its networking capability. Listening to the experiences of peers who struggle with the same challenges allowed participants to realise they were not alone, incompetent or to blame for difficult cases’. They further report that ‘being able to talk to colleagues who ‘get it’ was noted as an important means of enhancing professional self-care that helped participants cope with the emotional aspects of dementia care, increased their self-efficacy for this care and reinforced a sense of mission.’ These soft benefits have consistently been reported in evaluating the Africa Cancer ECHO and other ECHO programs [12] and are crucial given that African cancer care providers report high rates of job dissatisfaction and risk burn-out [10].

This study faced similar limitations as those reported in the literature for evaluating ECHO and similar initiatives [20]. These included a small sample size of only 38 respondents for the survey (response rate of 19.5%) with a risk of response and social desirability bias. Moreover, the assessment was done at the end of the curriculum, and respondents were expected to reflect on all the 12 sessions in a year. Since immediate (i.e., *Reaction*) outcomes such as satisfaction vary per session [22], the responses are subject to recall bias.

Another challenge is related to the nature of the Africa Cancer ECHO in that it deals with a variety of cancer control issues, as opposed to the clinical management of a specific disease (such as hepatitis C in Arora’s *et al* [13] original ECHO). The participants have varying levels of interest in the different sessions depending on their line of work, e.g., cancer research, advocacy, financing, information systems or treatment. It is, therefore, unlikely that an individual participant will attend all the sessions in the curriculum. Nonetheless, one-third of the 200 unique participants in the 2022/2023 curriculum attended at least two sessions, which suggests that some participants work in or are interested in many aspects of cancer control or that they are committed members of the VCoP. This could, however, lead to biased evaluation by participants who attended only a few sessions in the year, for example, by rating the sessions in their line of work more positively.

## Recommendations

Here we list some recommendations for future operation and evaluation of the Africa Cancer ECHO:

- Improve timing and frequency of evaluations: Evaluations should be made regularly after each session to capture transient and session-dependent outcomes (participation, satisfaction and so on), in addition to the long-term outcomes, such as behavioral changes and new, tangible initiatives.
- Monitor additional outcomes: Other measures and outcomes, such as engagement (e.g., the number of questions asked in a session), should be evaluated to get a more nuanced understanding of participation and interest in individual sessions. Interprofessional networking and peer-learning outcomes have also been proposed as long-term outcomes [12, 28], including identity, belonging and flattening of hierarchy (do participants feel more collegial? Is there level ground between ‘experts’ at the ‘hub’ and the ‘non-experts’ in the ‘spokes’?); increased trust, respect, prestige among participants; participants helping each other (or asking for help) for example in searching for jobs or education.
- Experimental designs: If possible, methods to get feedback from non-attendees or other control groups should be explored. Moreover, experimental designs that help to ascertain the impact of the ECHO and control for confounders (e.g., cluster randomised trials, RCTs) should be employed. In a study in Cameroon, a pre-post evaluation was used, which allowed evaluation of the impact of ECHO on cervical cancer prevention practitioners’ knowledge [29]. A few RCTs with different ‘doses’ of ECHO have been conducted but did not show differences between the groups [30–32], suggesting more robust evaluations are needed to strengthen the evidence base for ECHO programs.
- Flexibility: Adaptations to the needs of the members of the VCoP, as well as methods to encourage attendance, are needed, particularly for new members who might not be as committed and motivated as the core (steering committee) members. With many competing priorities and meeting fatigue, the Africa Cancer ECHO should make it as easy and rewarding for attendees. Over the years, the Africa Cancer ECHO has adapted the curriculum to participant input, for example, by increasing the session duration to 90 minutes in 2022/2023 after participants indicated insufficient time for discussion in the prior evaluations. However, a few suggestions that could attract participants have yet to be implemented, e.g., issuing continuous professional development credits, providing opportunities for physical meetings and so on. Some of these require financial and human resources to implement, and without dedicated funding for the ECHO, addressing them will remain a challenge.
- Marketing and expansion: There are also opportunities to expand the community through this continuous adaptation as well as marketing, for example, by sharing on social media platforms WhatsApp, LinkedIn and X, which are widely used in Africa. Currently, recorded sessions are shared on YouTube, but no live streaming is done. Rather, attendees have to register in advance via the iECHO platform, which is an extra step that can deter them from joining. Participating in and advertising at conferences and community events related to cancer is another avenue for expanding and potentially increasing impact. At the same time, time and effort are needed to continue cultivating the shared identity and intent [11], which is currently done by the voluntary steering committee and KENCO.
- Additional languages: The addition of sessions in other major languages spoken in Africa, e.g., French, Arabic and Portuguese (or live translation to these languages during the sessions), could potentially improve attendance from other countries.

## Conclusion

VCoPs, such as the Africa Cancer ECHO, have the potential to contribute to human resource capacity-building for cancer control in Africa through accessible and convenient peer learning, professional networking and collaboration. However, identifying and using more robust evaluation tools and methods may provide a more comprehensive assessment of all of their benefits, including short and long-term outcomes.

## Conflicts of interest

The authors declare no conflicts of interest.

## Funding

This study was funded by the Stockholm University Library.

## Figures and Tables

**Table 1.** Africa cancer ECHO 2022–2023 session details.

Date	# attendees	NCCP core element, based on Oar et al [14]	Topic	Case presenter organization, country	Didactic presenter organization, country
September 15 2022	49	Prevention	The role of HPV DNA testing in fast-tracking cervical cancer prevention in Africa	Teal Sisters Foundation, Zambia	College of Medicine, University of Ibadan, Nigeria
October 20 2022	27	Diagnosis, staging, and screening	Use of telepathology to advance and streamline quality cancer diagnostics as part of the cancer continuum pathway	Mbingo Baptist Hospital, Cameroon	International Cancer Institute, Kenya
November 17 2022	40	Treatment	Cancer care and management in LMICs – addressing access and affordability of essential medicines	Princess Marina Hospital, Ministry of Health and Wellness, Botswana	University of Pennsylvania, USA
December 15 2022	28	Palliative care and survivorship	Integration of survivorship at the policy level in Africa	Panel Discussion Aga Khan University Hospital, Kenya Center for Global Health, National Cancer Institute, US Department of Psychology, Maynooth University, Ireland/Kenya Uganda Child Cancer Foundation, Uganda Uganda Women’s Cancer Support Organization, Uganda	
January 19 2023	27	Health workforce	Strengthening service delivery through patient navigation for improved cancer outcomes in LMICs	Women’s Coalition Against Cancer (WOCACA), Malawi	National Cancer Control Unit, Ministry of Health, Eswatini
February 16 2023	26	Financing	Navigating public private partnerships to leverage opportunities while promoting equity and sustainability	Non-Communicable Diseases Division, Rwanda Biomedical Centre/Ministry of Health, Rwanda	Commission of Health and Human Services, Ekiti State, Nigeria
March 16 2023	27	Health workforce	Sensitization of primary HCPs	International Training and Education Center for Health, Namibia	Jhpiego, Tanzania
April 20 2023	37	Health information systems	Health information Systems – from the individual to aggregate level	National Cancer Registry of South Africa and IARC-GICR Collaborating Centre, South Africa	Brown University, Providence, RI, USA
May 2023	39	Research	Research authorship: strengthening representation of African researchers and program implementers in global oncology journals	Harvard Medical School and Dana-Farber Cancer Institute, USA JCO Global Oncology and National Center for Radiotherapy, Oncology and Nuclear Medicine, Korlebu Teaching Hospital, Ghana eCancer Medical Science (UK), The Oncologist (USA), and Latin America and Caribbean Society of Medical Oncology, Argentina PLOS Global Public Health and Institute of Human Virology, Nigeria	
June 15 2023	27	Health workforce	Integration of cervical cancer screening and primary care capacity building (from health workforce, cancer advocacy, and policy perspectives)	Ministry of Health, Ethiopia Clinton Health Access Initiative, Zambia	National Cancer Control Programme, Ministry of Health, Kenya
July 20 2023	34	Financing	Defining the factors that create financial barriers to care	Federal Ministry of Health, Ethiopia	Kenya Medical Research Institute and RTI International, Kenya
August 2023	36		5-year retrospective of Africa Cancer ECHO.	Africa ECHO Steering committee	

**Table 2.** Joint publications from the Africa cancer ECHO.

Author and Year	Tittle of the publication	Aim of the study	Main results and recommendations
Duncan *et al* 2019 [15]	Use of telementoring to advance cancer control: The 2018 Africa Cancer Research and Control ECHO^®^ Programme	To measure the impact and contribute to the evidence about the potential effectiveness of ECHO in strengthening NCCP and implementation	48 participants from 12 countries registered for the Africa ECHO. 95% reported that their cancer control-related challenges had been addressed. 40% reported developing new collaborations The use of telementoring provides a platform for knowledge sharing and multi-directional learning to support the implementation of NCCPs for countries and international partners alike.
Nakaganda *et al* 2021 [16]	Expanding best practices for implementing evidence-based cancer control strategies in Africa: the 2019-2020 Africa Cancer research and control ECHO program	Aimed to increase knowledge and utilization of evidence-based practices to strengthen NCCPs in Africa through online monthly hour-long sessions about cancer control.	157 participants registered for the Africa Cancer ECHO. 24 sessions were conducted for the year 2019-2020 70% of the participants increased their knowledge, confidence, and ability to implement evidence-based cancer control strategies in their settings. Over 80% indicated that sessions were relevant to their work and met their learning goals and expectations. The 2019–2020 Africa Cancer ECHO recommends use of data, partnerships, and locally-driven solutions to direct the cancer control effort in Africa.
Nakaganda *et al* 2023 [17]	How COVID-19 exposed pre-existing roadblocks for cancer control in Africa: strategies, lessons and recommendations from the 2019-2020 Africa Cancer Research and Control ECHO	Described the evolved strategies, dilemmas, and recommendations to strengthen the health systems for cancer control in Africa	Most strategies to maintain cancer services during the COVID-19 pandemic centered around cancer treatment. This analysis shows that the COVID-19-related mitigation measures exacerbated existing predicaments in Africa, such as inadequate attention to cancer prevention strategies, psychosocial/palliative services, and cancer research. African countries should leverage the infrastructure and information technology developed in response to the COVID-19 pandemic to strengthen the health system along the entire cancer control continuum and to develop or implement evidence-based NCCP that will withstand any future disruptions of cancer services.
Lasebikan *et al* 2022 [18]	Demonstration of impact through the evaluation of the 2020–2021 Africa Cancer Research and Control ECHO	The 2020-2021 evaluation included for the first time a qualitative component, allowing documentation of concrete utilization of knowledge gained from the Africa Cancer ECHO to advance cancer control efforts.	226 unique people from over 40 organizations participated in the Africa Cancer ECHO during 2020–2021 19 sessions were hosted on topics across the cancer control continuum. 100% strongly agreed that the sessions were relevant and engaging. knowledge gained was applied to add survivorship to their NCCP (for the first time); adapting training materials to initiate a patient navigation program, partnering to strengthen brachytherapy capacity at the national level, and collaboratively conducting a situational analysis of cancer survivorship in the Africa region. reported limitations included obstacles of time (74%), technology (13%), and limited opportunities to engage with partners and participants (50%). Recommended more targeted recruitment and retention, as well as facilitation of collaborations beyond the sessions, will further strengthen this community.
Garton *et al* 2023 [25]	An analysis of survivorship care strategies in NCCP in Africa	Examined the inclusion of cancer survivorship-related strategies and objectives in NCCPs of African countries.	21 NCCPs were examined. In total there were 202 survivor-related strategies and all NCCPs included a range of 1 and 23 survivorship strategies Only 41% of the strategies were linked to measurable indicators Most (63%) of the survivorship strategies were focused on palliative care only. NCCP Survivorship strategies should extend beyond palliative care to encompass all aspects of survivorship and should include indicators to measure progress.

## Appendix 1: survey responses

**Table:**

		Year			
Variable/Characteristis (n, %)		2019-2020	2020-2021	2021-2022	2022-2023
	Total (n)	38	22	37	38
					
Countries representated	Burundi	2	2	0	2
	Cameroon	2	0	2	2
	Cote d'Ivoire	0	0	1	1
	Eswatini	1	1	0	1
	Ethiopia	0	3	1	2
	Ghana	0	0	1	0
	Kenya	17	8	13	13
	Malawi	0	0	2	5
	Namibia	0	0	0	1
	Nigeria	2	2	3	2
	Rwanda	1	0	1	0
	South Africa	1	0	0	1
	Tanzania	1	0	0	1
	Uganda	3	2	2	2
	Zambia	1	1	2	2
	Zimbabwe	2	2	4	2
	Multiple African Countries	0	0	2	0
	Country outside Africa	5	1	3	1
**Cancer control domain (n, %)**	Cancer NGO/Advocacy	14	11	17	13
	Clinician	5	3	8	12
	Researcher	8	4	2	3
	Healthcare administration (Ministry of Health, etc)	11	4	9	8
	Patient			1	2
**Participation**					
Year of joining	Joined that year	14	8	13	8
	Returning from previous years	24	14	24	30
**Attendance**	Regularly (every or almost every session)	20	11	18	20
	Occasionally (once every 2-3 months, approx.)	14	9	15	11
	Seldom (1-2 times)	3	2	4	7
	Never	1	0	0	0
**Reaction**					
The topics covered are relevant to my work	Agree	35	21	33	34
	Partially Agree	3	1	4	4
	Disagree	0	0	0	0
The speakers provide insights that are relevant to my work	Agree	0	0	34	35
	Partially Agree	0	0	3	3
The sessions were engaging and worth my time	Agree	36	22	35	35
	Partially Agree	2	0	2	2
	Disagree	0	0	0	1
The structure accommodates my learning style	Agree	33	17	30	28
	Partially Agree	5	5	7	9
The ECHO met my learning goals and expectations.	Agree	31	16	28	27
	Partially Agree	7	6	9	11
	Disagree				
**Learning**					
I now possess relevant knowledge about cancer control principles and strategies after attending the Africa ECHO.	Agree	30	17	25	26
	Partially Agree	8	5	12	12
I have gained confidence in planning and implementing evidence-based cancer control strategies by attending the Africa ECHO sessions.	Agree	26	13	22	22
	Partially Agree	12	9	15	16
**Networking/Community**					
I feel confident to professionally interact with colleagues in cancer control	Agree	30	18	27	36
	Partially Agree	8	4	10	6
As a result of participation in the Africa ECHO, I have ideas on how and where I can find support for dilemmas in cancer control from subject matter experts within Africa and internationally.	Agree	22	17	27	27
	Partially Agree	16	5	10	11
As a result of participation in the Africa ECHO, I feel a sense of belonging to a community/team of cancer control leaders in the region and internationally.	Agree	33	19	31	31
	Partially Agree	5	3	6	7
**Performance**					
I have put the knowledge I gained from the ECHO sessions to use	Agree	28	16	26	24
	Partially Agree	10	5	11	14
I am able to transfer the knowledge, skills or attitudes that I have gained from the ECHO to others.	Agree	31	15	27	28
	Partially Agree	7	7	10	10
**Meeting of objectives**					
The ECHO enabled disseminating best practices and engaging partners/participants in a continuous mentoring and learning system	Very much	29	14	26	28
	Somewhat	9	7	11	10
	Not at all	0	1	0	0
The ECHO increased knowledge and utilization of evidence-based cancer control principles and strategies	Very much	30	16	28	26
	Somewhat	8	5	9	12
	Not at all	0	1	0	0
The ECHO created opportunities to network and learn from regional and global colleagues and experts	Very much	28	13	25	30
	Somewhat	10	8	12	8
	Not at all	0	1	0	0
Have your interactions within Africa ECHO sessions led to any follow-up discussions and/or collaborations with fellow ECHO participants and/or speakers?	Yes	*	11	21	22
	No	*	11	16	16
**Obstacles to attendance**	Connectivity issues	*	3	6	13
*Note: More than 1 option possible, so total can be more that 100%*	Other pressing/competing commitments	*	16	23	26
	Session length (90 minutes)	*	0	0	2
	Timing of the sessions (time of day, day of week)	*	0	7	13
	Forgetting about the sessions	*	0	0	0
	Relevance of the topic	*	0	1	1
*Question not asked this year					
